# Lysins as a powerful alternative to combat *Bacillus anthracis*

**DOI:** 10.1007/s00253-024-13194-3

**Published:** 2024-06-08

**Authors:** Aleksandra Nakonieczna, Karolina Abramowicz, Magdalena Kwiatek, Ewelina Kowalczyk

**Affiliations:** 1 Military Institute of Hygiene and Epidemiology, Biological Threats Identification and Countermeasure Center, Puławy, 24-100 Poland; 2 National Veterinary Research Institute, Puławy, 24-100 Poland

**Keywords:** Endolysin, *Bacillus anthracis*, Lytic activity, Anthrax, CBD domain

## Abstract

**Abstract:**

This review gathers all, to the best of our current knowledge, known lysins, mainly bacteriophage-derived, that have demonstrated activity against *Bacillus anthracis* strains. *B. anthracis* is a spore-forming, toxin-producing bacteria, naturally dwelling in soil. It is best known as a potential biowarfare threat, an etiological agent of anthrax, and a severe zoonotic disease. Anthrax can be treated with antibiotics (ciprofloxacin, penicillin, doxycycline); however, their administration may take up even to 60 days, and different factors can compromise their effectiveness. Bacterial viruses, bacteriophages (phages), are natural enemies of bacteria and use their lytic enzymes, endolysins (lysins), to specifically kill bacterial cells. Harnessing the potential of lysins to combat bacterial infections holds promise for diminishing antibiotic usage and, consequently, addressing the escalating antibiotic resistance in bacteria. In this context, we list the lysins with the activity against *B. anthracis*, providing a summary of their lytic properties in vitro and the outcomes observed in animal models. *Bacillus cereus* strain ATCC 4342/RSVF1, a surrogate for *B. anthracis*, was also included as a target bacteria.

**Key points:**

• *More than a dozen different B. anthracis lysins have been identified and studied.*

• *They fall into three blocks regarding their amino acid sequence similarity and most of them are amidases.*

• *Lysins could be used in treating B. anthracis infections.*

## Introduction

Natural infections caused by *Bacillus anthracis*, an etiological agent of anthrax, are not common. However, the general knowledge makes average people aware of this bacteria as a potential biowarfare threat and a tool of bioterrorists. According to ECDC (http://atlas.ecdc.europa.eu/public/index.aspx, accessed on 13 Jan 2024), 30 natural cases of anthrax were reported in the countries of the EU within the 2017–2022 period. No data on recent worldwide case reports from WHO and CDC is available; however, an annual global incidence of 2000–20,000 cases in the twenty-first century is estimated by the WHO (Simonsen and Chatterjee 2022). The disease is a significant public health problem in Central Asia and Africa (Ozer et al. 2019; Nakanwagi et al. 2020). In nature, people get infected primarily from grazing animals, and some occupations are especially at risk (butchers, vets, tanners, wool sorters, scientists). Inhalational anthrax onset resembles flu but can develop severe pneumonia-like symptoms and cause high mortality if not treated immediately. The gastrointestinal form of anthrax is also severe, carrying a 40% mortality rate even with treatment (Hendricks et al. 2014). In contrast, cutaneous anthrax accounts for 95% of all cases, being the least hazardous and tending to self-healing within 2–3 weeks in uncomplicated cases (Ozer et al. 2019). A new clinical form of anthrax is caused by injection of spore-contaminated heroin (9–33% mortality; Booth et al. 2014; Hendricks et al. 2022). While anthrax can be effectively treated with readily available antibiotics, the escalating challenge of antimicrobial resistance (AMR) has spurred a scientific exploration into alternative approaches for combating bacterial infections. Such approaches are, for instance, the use of plant compounds (Dassanayake et al. 2021), as well as bacterial viruses known as bacteriophages (phages) and phage-encoded lytic enzymes directly responsible for killing the bacterial cells, endolysins (shortly called lysins). Bacteriophages and their lysins have been studied for decades and demonstrate certain advantages over antibiotics (Liu et al. 2023). Lysins can be easily produced as purified proteins, and many of them have proved their effectiveness against different bacteria species in vitro and in vivo. These highly specific lytic enzymes target crucial peptidoglycan bonds necessary for maintaining its structural integrity. In terms of their enzymatic domain activity, which dictates the type of chemical bond they cleave, lysins are classified into three primary classes, with the respective cleavage sites: *N*-acetylmuramoyl-l-alanine amidases (cleave bonds between the sugar and amino acid moieties), endopeptidases (cleave peptide bonds between amino acids), and glycosidases (cleave glycosidic bonds between MurNAc and GlcNAc) (Abdelrahman et al. 2021). Glycosidases are further subdivided into *N*-acetyl-β-d-muramidases (lysozymes), lytic transglycosylases, and *N*-acetyl-β-d-glucosaminidases (glucosidases). The fact that bacteria do not develop resistance to lysins is their crucial asset (Gondil et al. 2020; Abdelrahman et al. 2021; Arroyo-Moreno et al. 2022). Lysozymes and amidases are the predominant lysins in Gram-negative and Gram-positive bacteria-infecting phages, respectively (Vázquez et al. 2021).

Currently, 41 complete genomes of *B. anthracis* phages are deposited in the NCBI database (https://www.ncbi.nlm.nih.gov/, accessed on January 12, 2024). Although this number is comparatively lower than that for some more prevalent bacterial species, it stands relatively high for bacteria characterized by long dormancy periods due to spore-forming capabilities and, consequently, low genetic variability. Among these phages, only a subset of lysins has undergone characterization and study. While certain lysins, such as those from phages Gamma, Wbeta, Fah, AP631, and Cherry, are identical, others exhibit greater diversity and possess distinct enzymatic activities. So far, selected lysins active against *B. anthracis* were usually mentioned in various reviews alongside other G-positive bacteria lysins or as random lysins examples. This mini-review constitutes a first concise overview of all identified *B. anthracis* lysins, describing their lytic properties together with their cell wall–binding domain comparison. All information is lucidly shown in tables showing the lysins host ranges within the *Bacillus cereus* group and the main outcomes of their use in animal models.

## Antibiotic resistance in *B. anthracis*

Current approaches to anthrax treatment rely on the administration of broad-spectrum antibiotics (ciprofloxacin, penicillin, and doxycycline) alongside antitoxin therapy. However, there are some concerns about the use of antibiotics in this case, such as the time-consuming determination of bacterial susceptibility, as observed with penicillin (Brook 2002), poor penetration of β-lactam antibiotics (penicillin) into macrophages where the spores germinate (Bell et al. 2002), production of penicillinase and cephalosporinase (β-lactamase) (Leonard et al. 2021), and the potential susceptibility to engineered resistance (Klimko et al. 2022). An antibiotic that turned out to be highly effective against *B. anthracis* is also novobiocin, an underused, early generation aminocoumarin produced by *Streptomyces niveus*. It was found to require low concentrations to effectively kill different biowarfare agents in mouse models (Klimko et al. 2022), and it could be an additional approach among already established drugs for treating anthrax.

Recently, a large-scale analysis of a global collection of 356 *B. anthracis* genomes has revealed the presence of ten AMR genes with five of them being notably widespread across the majority of examined isolates (Bruce et al. 2021). Although the presence of such genes does not always translate to actual resistance (Bruce et al. 2021), it underscores the importance of alerting medical professionals to the potential emergence of a significant treatment challenge. Monitoring the occurrence of rare *B. anthracis* AMR genes in specific regions is crucial for pinpointing areas where the situation is particularly problematic and may necessitate more concerted efforts in treatment protocols (Bruce et al. 2021). Interestingly, it was observed that the use of a chemical mutagen substantially increased the frequency of antibiotic resistance in *B. cereus* RSVF1. However, this manipulation did not alter the sensitivity of the bacteria to PlyG, the best-known *B. anthracis* lysin (Schuch et al. 2002).

## In vitro studies of the lysins’ activity

The lysins’ antimicrobial activity was tested on various bacterial species. Table 1 displays the host ranges of lysins among the members of the *Bacillus cereus* group that encompasses *B. anthracis*, *B. cereus*, *B. thuringiensis*, *B. mycoides*, and *B. weihenstephanensis* species. Some laboratories, due to the lack of a vaccine strain (*B. anthracis* Sterne 34F2) or virulent anthrax strains, or for safety reasons, use surrogate strains instead as they can serve as suitable representatives. The closest safety-providing homolog and best representative of *B. anthracis* properties for lab work purposes is *B. cereus* RSVF1, a streptomycin-resistant derivative of plasmid-free *B. cereus* ATCC 4342 (Schuch et al. 2002; Severin et al. 2004). *B. cereus* RSVF1 and *B. cereus* ATCC 4342 are closely related genetically, morphologically, and physiologically to *B. anthracis*; they all are sensitive to Gamma phage and its lysin PlyG; thus, these two can be used as acceptable surrogates of virulent anthrax strains in the lysin studies (Schuch et al. 2002; Severin et al. 2004; Porter at al. 2007). In Table 1, we included one lysin, Ply57, whose activity was not tested against *B. anthracis* strains, but *B. cereus* ATCC 4342 was used instead. Additionally, lysins LysJ and LysF were tested on “transitional” strains belonging to the *B. cereus* group that are plasmid-free but encode a chromosomal anthrax marker gene, *Ba 813*, i.e., *Bacillus* sp. *Ba 813+* (Niemcewicz and Bartoszcze 2006).

**Table 1:** Summary of in vitro activity assays of phage lysins or lysins of phage origin lytic against *B. anthracis*. Cases where *B. cereus* RSVF1/ATCC 4342 was used, but not anthrax strains, are also included

	Lysin	Phage	Phage host bacteria	Lysin enzymatic activity	Lysin length (aa)	Lysin host spectrum (only bacteria from the *B. cereus* group are listed)	Lytic activity assay	References
1.	**LysJ** (QOQ37201.1)	J5a	*B. anthracis* Sterne 34F2	Amidase	324	***B. anthracis***: Sterne 34F2, 211, 1153, 1583, 1584, PZH *B. cereus*: ATCC 10,872, ATCC 10,876, ATCC 11,778, ATCC 13,472, ATCC 14,579^T^, ATCC 19,637, ATCC 23,261, F16959, F17202, F17289, UW85 *B. thuringiensis*: ATCC 33,679, ATCC 35,646, ATCC 10,792, ATCC 10,792^T^, T07-019, T07-128, T07-146, T07-151, T07-155, T07-202, #35 *B. mycoides*: ATCC 6462, ATCC 21,929, K184 *B.* *sp.* *Ba 813+*: #6 (I/2), #7 (II/3), #12 (S8553/2), #16 (PJ572), #17 (094), #21 (T1197-77), #28 (3), #30 (1B), #31, #3403	Turbidity reduction/spot test	Nakonieczna et al. 2024
2.	**LysF** (QOQ37151.1)	F16Ba	*B. anthracis* Sterne 34F2	Amidase	324	***B. anthracis***: Sterne 34F2, 211, 1153, 1583, 1584, PZH *B. cereus*: ATCC 10,872, ATCC 10,876, ATCC 11,778, ATCC 13,472, ATCC 14,579^T^, ATCC 19,637, ATCC 23,261, F16959, F17202, F17289, UW85 *B. thuringiensis*: ATCC 33,679, ATCC 35,646, ATCC 10,792, ATCC 10,792^T^, T07-019, T07-128, T07-146, T07-151, T07-155, T07-202, #35 *B. mycoides*: ATCC 6462, ATCC 21,929, K184 *B.* *sp.* *Ba 813+*: #6 (I/2), #7 (II/3), #12 (S8553/2), #16 (PJ572), #17 (094), #21 (T1197-77), #28 (3), #30 (1B), #31, #3403	Turbidity reduction/spot test	Nakonieczna et al. 2024
3.	**LysPW2** (AZU98917.1)	PW2	*B. cereus*	Amidase	307	***B. anthracis***: Sterne 34F2 ***B. paranthracis***: EFR-1, EFR-4, EFR-5 *B. cereus*: F4810/72, NC7401, 5975 C, IS075, LH001, EFR-2 ATCC 7953, AND1407, ATCC 10,987, ISP2954, ATCC 14,579, ISP3191 *B. thuringiensis*: BMB171, ATCC 35,646, HD-73, GPL-O901 *B. weihenstephanensis*: CER057, MC67, KBAB4	Turbidity reduction	Wan et al. 2021
4.	**Ply57** **(**QIW89878.1)	Izhevsk	*B. cereus* VKM B-370	Amidase	313	*B. cereus*: **ATCC 4342**, ATCC 14,893, VKM: B-13, B-370, B-373, B-374, B-383, B-445, B-473, B-491, B-504^T^, B-681, B-682, B-683, B-684, B-686, B-688, B-771, B-810, B-812 *B. thuringiensis*: ATCC 35,646, VKM: B-83, B-84, B-85, B-440, B-443, B-446, B-447, B-450, B-453, B-454, B-1555, B-1557 *B. weihenstephanensis*: KBAB4	Turbidity reduction	Skorynina et al. 2020
5.	**LysBC17** (QEM39038.1)	Probably retained in *B. cereus* genome from a phage	*B. cereus* Bc17	Peptidase	289	***B. anthracis***: Ames 35, UM23 *B. cereus*: Bc17, **ATCC 4342**, ATCC 11,778, ATCC 13,061, ATCC 14,579 *B. thuringiensis*: ATCC 13,061	Spot test	Swift et al. 2019
6.	**PlyP56** (AMW62097.1)	Phrodo	*B. thuringiensis* subsp. *kurstaki*	Peptidase	259	***B. anthracis***: Ames 35, UM23 *B. cereus*: **ATCC 4342** *B. cereus*: **ATCC 4342**, ATCC 14,579, ATCC 11,778, ATCC 13,061 *B. thuringiensis*: ATCC 10,792	Spot test Turbidity reduction	Etobayeva et al. 2018
7.	**PlyN74** (AMW61226.1)	Nigalana	*B. thuringiensis* subsp. *kurstaki*	Amidase	275	***B. anthracis***: Ames 35, UM23 *B. cereus*: **ATCC 4342** *B. cereus*: **ATCC 4342**, ATCC 14,579, ATCC 11,778, ATCC 13,061 *B. thuringiensis*: ATCC 10,792	Spot test Turbidity reduction	Etobayeva et al. 2018
8.	**PlyTB40** (ALA13156.1)	TsarBomba	*B. thuringiensis* subsp. *kurstaki*	Amidase	272	***B. anthracis***: Ames 35, UM23 *B. cereus*: **ATCC 4342** *B. cereus*: **ATCC 4342**, ATCC 14,579, ATCC 11,778, ATCC 13,061 *B. thuringiensis*: ATCC 10,792	Spot test Turbidity reduction	Etobayeva et al. 2018
9.	**AP50-31** (YP_002302537.1)	AP50	*B. anthracis*	Amidase	252	***B. anthracis***: ΔSterne, Sterne 34F2, ATCC 14,578, HYU01 *B. cereus*: **ATCC 4342** *B. thuringiensis*: BGSC 4AJ1, BGSC 4BA1, BGSC 4CC1	Turbidity reduction	Park et al. 2018
10.	**gp217** (AGI11763.1)	Tsamsa	*B. anthracis*	Amidase	313	***B. anthracis***: 6602 R1, Sterne 34F2, Weybridge UM44, Ames-non reverting *B. cereus*: LA 925, ATCC 14,579, ATCC 11,778, ATCC 10,702, DSM 2302, DSM 4218, ATCC 33,019, ATCC 14,737, DSM1274, ATCC 27,522, NCTC 11,143, NCIMB 8705, ATCC 6464, B346, HER1399, WSBC 10,556 *B. thuringiensis*: DSM4421, HER1211, ATCC 10,792 *B. weihenstephanensis*: WSBC 10,550	Turbidity reduction/spot test	Ganz et al. 2014
11.	**PlyBt33** (AFL46409.1)	BtCS33	*B. thuringiensis*	Amidase	272	***B. anthracis***: CMCC63605 *B. cereus*: strains not specified *B. thuringiensis*: subsp. *kurstaki*, HD-73, HD-1	Turbidity reduction	Yuan et al. 2012
12.	**LysB4** (YP_006908235.1)	B4	*B. cereus*	Peptidase	263	*B. cereus*: ATCC 40,133, ATCC 27,348 ***B. anthracis***: ΔSterne, Sterne, ATCC 14,578, HYU01 *B. cereus*: **ATCC 4342** *B. thuringiensis*: BGSC 4AA1	Turbidity reduction	Son et al. 2012 Park et al. 2018
13.	**PlyB** (YP_009031336)	Bcp1	*B. anthracis* RS222 (*B. cereus* according to NCBI)	Lysozyme	284	*B. cereus*: **ATCC 4342** ***B. anthracis***: Sterne 34F2, ΔSterne, RS222 (Spo−, ΔSterne) *B. cereus*: **ATCC 4342** (γ-sensitive), E33L (Zebra killer), CDC 32,805 (γ-sensitive), CDC 13,100, CDC 13,140, ATCC 10,987, ATCC 14,579, ATCC 11,950, ATCC 13,472, ATCC 9592, ATCC 25,621, ATCC 43,881, ATCC 14,893, ATCC 27,877, ATCC 7064, T, NRRL 569, RTS 134, RTS 100, HER 1414, DP.B4833 (UM101), DP.B5129 (UM101:: ϕZ1), DP.B5184 (UM101::ϕZ3), DP.B5185 (UM101:: ϕZ4), DP.B5208 (S58), DP. B5209 (UM101:: ϕS58), BGSC 6A6, BGSC 6E1, 03BB108, AH187, D-17, D-33, 3 A, 03BB87, S2-8, 03BB102, NE-chicken, F4431/73, F4433/73, F1589/77, FM-1, F2105/89, F837/76, F3748/75 *B. thuringiensis*: 97–27 (human, necrosis), Al Hakam, HD-73 *kurstaki*, HD-1 *kurstaki*, HD-4 *alesti*, HD-30 *canadensis*, HD-135 *aizawai*, HD-453 *kenyae*, HD-538 *tolworthi*, HD-571 *kyushuensis*, HD-682 *finitimus*, HD-819 *yunnanensis*, HD-866 *tohokuensis*, HD-868 *tochigiensis*, HD-974 *nigeriae*, HD-1011 *pondicheriensis*, DP.B5135 *brasiliensis*, DP.B5132, BGSC 4BR1 *poloniensis*, BGSC 4BU1 *pirenaica*, BGSC 4I1 *entomocidus*, BGSC 4J1 *pacificus*, DP.B5198, DP.B5199, HER 1410 *B. mycoides*: BGSC 6A13, BGSC 6A19, ATCC 6462, ATCC 23,258, BGSC 6A11, BGSC 6A12, BGSC 6A14	Turbidity reduction	Porter et al. 2007 Schuch et al. 2019
14.	**PlyPH** (WP_000540633.1)	Putative phage origin (ORF BA2805 in *B. anthracis* Ames)	*B. anthracis* Sterne 34F2	Amidase	268	***B. anthracis***: ΔSterne *B. cereus*: **RSVF1**, ATCC 10,987 *B. cereus*: **ATCC 4342**	No data available Turbidity reduction	Yoong et al. 2006 Bhagwat et al. 2021
15.	**PlyL** (WP_000405801.1)	Prophage Lambda Ba02	*B. anthracis* Ames	Amidase	234	***B. anthracis***: Sterne 34F2 *B. cereus*: **ATCC 4342**	Turbidity reduction	Low et al. 2005
16.	**PlyG** (YP_338200.1)	Gamma	*B. anthracis*	Amidase	233	***B. anthracis***: Vollum, Ames, A1.a/10, A1.b/23, A2//29, A3.a/34, A3.b/57, A4/69, B/80, ΔSterne, VNR1Δ1, ΔAmes, NNR1Δ1, ΔNH1 *B. cereus*: **RSVF1**, ATCC 10,987	Spot test Time-kill assay	Schuch et al. 2002

Most lysins with activity against *B. anthracis* are *N*-acetylmuramoyl-l-alanine amidases, three are peptidases, and one has an activity of lysozyme (Table 1). Enzymatic activity of all described lysins except one was studied on at least one anthrax strain, and most of them were also tested on *B. cereus* ATCC 4342/RSVF1. While most lysins in Table 1 are typical phage enzymes, some were found in the bacterial DNA. LysBC17, for instance, lacks a typical phage origin. However, its gene likely persisted in bacterial genomes following a previous infection with a temperate phage, with subsequent loss of most prophage sequences over time (Swift et al. 2019). LysBC17 resembles the domain architecture and sequence of other *Bacillus* phage lysins. Hence, its bacteriolytic properties were included in this review. PlyPH lysin has a putative phage origin but was found in a few *B. anthracis* strains by BLAST search and PCR amplified from *B. anthracis* 34F2. It was suggested as a lysin or a close relative of phage lysins due to its high identity results against various *Bacillus* phages, including *B. anthracis* prophages (Yoong et al. 2006). Another example is PlyL from the *B. anthracis* λ prophage Ba02. This amidase shares the most similarity with PlyG in both the enzymatic (93% identity) and binding (60% identity) domains (Low et al. 2005). What is interesting about this enzyme is the fact that its enzymatic activity domain (EAD), PlyL_CAT_, exhibited greater efficacy in lysing *B. anthracis* cells compared to the full-length protein (Low et al. 2005). Regarding the domains, it is worth recalling another example, AP50-31 lysin, which has only a catalytic domain and lacks a cell wall–binding domain (CBD) that determines the host range for the enzyme. It still caused a rapid and effective bacteriolytic effect comparable to that of LysB4, a classical two-domain potent *B. anthracis* lysin, evaluated along with AP50-31 in one study (Park et al. 2018). For another lysin, PlyB, the full enzyme activity measured on *B. cereus* ATCC 4342 was as strong as that of PlyG against the same strain, but both domains were shown to be equally required for effective killing. Only after 2 h could PlyB EAD alone cause complete lysis (Porter et al. 2007). Interestingly, it was reported that the charge of the EAD correlates with its activity and that truncated, positively charged EADs demonstrate better enzymatic activity than full-length proteins (Low et al. 2011). This is consistent with the results obtained, e.g., for positively charged PlyG_CAT_ and PlyL_CAT_ and negatively charged PlyB_CAT_ (Low et al. 2011). It is not rare that the presence of CBD is essential for the whole protein activity, like also in the case of PlyBt33, where the catalytic domain alone had relatively low activity, or for LysBC17_CAT_ and LysPW2_CAT_, where no activity was detected. However, what should be highlighted, it was found that LysPW2 could bind vegetative cells and spores of *B. thuringiensis* EFR-4, and the LysPW2_CAT_ domain inhibited 66.3% spore germination (Wan et al. 2021).

Two assays are routinely performed for lysins activity determination, spot test (spot assay, plate lysis assay) and turbidity reduction assay (optical density (OD) reduction). The spot test is usually a simple experiment to determine the bacterial spectrum of lytic activity. In contrast, OD reduction better shows the actual level of this activity against bacterial cells in suspensions. For recently published LysJ and LysF, the latter method proved to be more sensitive since both lysins demonstrated good killing activity in liquid suspensions of most of the tested strains from the *B. cereus* group, including all *B. anthracis* strains, but only the anthrax strains were susceptible in the spot assay (Nakonieczna et al. 2024). On the other hand, contrasting results were obtained for PlyP56 and PlyN74, which caused significant lysis of *B. anthracis* strains in plates but not in OD reduction assay (Etobayeva et al. 2018). Most of the presented lysins have broad-spectrum activity against *B. cereus sensu lato* or more extensive against the *Bacillus* genus. High specificity against *B. anthracis* strains or *B. cereus* RSVF1 is shown only for PlyG and PlyPH. These lysins share a high CBD sequence identity, which may implicate a comparable host range. Additionally, the determination of the lytic activity against another genus (including Gram-negative bacteria) that was performed for gp217, LysPW2, LysBC17, PlyBt33, PlyL, and PlyB revealed their specificity exclusively to the *Bacillus* genus.

## In vivo studies of the lysins’ activity

Once the high antimicrobial activity of a lysin under in vitro conditions is proven, it is advisable to conduct in vivo studies. Animal models play a crucial role in confirming the efficacy and assessing the safety of lysin treatments in complex organisms. In Table 2, we have summarized the in vivo tests that were conducted so far for four lysins: PlyG, PlyPH, PlyB, and LysB4. Three *Bacillus* strains were used in these studies: *B. cereus* RSVF1 (intraperitoneally and intravenously), *B. anthracis* ΔSterne (pX01^-^/pX02^-^; intravenously), and *B. anthracis* Sterne 34F2 (pX01^+^/pX02^-^; intranasally). The mouse model was chosen for all experiments (Table 2). The murine model is commonly applied in in vivo studies because of its susceptibility to lethal infections caused by attenuated strains. As they do not have a capsule but can produce toxins (*cap*-, *tox*+), they can still cause anthrax which is associated with high mortality and severe inflammation in mice (Welkos et al. 2016). The lysins were administered via intraperitoneal, intranasal, or intravenous routes (Table 2). An intraperitoneal injection is a commonly used infection route in rodent models because of its simplicity and minimal stress for mice. This method is justifiable in studies assessing the antimicrobial activity of lysins, supported by proof-of-concept studies aiming to determine the effect of target engagement (Al Shoyaib et al. 2020). For the lysin LysB4, the intranasal route was employed for both *Bacillus* infection and lysin application. This approach enhances the likelihood of direct contact between the lysin and infectious microorganisms. Furthermore, this method of infection best simulates inhalational anthrax (Park et al. 2018). *B. cereus* RSVF1 categorized as a BSL-1 microorganism was selected for safety reasons. Although studies with this strain are valuable, its mechanism of pathogenesis is entirely different (Park et al. 2018). RSVF1 strain, deprived of the pXO1 plasmid, does not produce anthrax toxins, which considerably affects the pathogenesis and inhibits the host’s effective immune response (Savransky et al. 2020). For PlyB and LysB4 studies, *B. anthracis* strains were applied (Table 2).

**Table 2:** Outcomes of *in vivo B. anthracis* lysins activity assays

Lysin	Target bacteria	Animal model	Route of lysin administration	Outcome	Reference
PlyB	*B. anthracis* ΔSterne (1 × 10^7^ CFU/ml; i.p.)	Mouse	i.v.	• Lysin was injected 1 h post-infection in 100 µl doses • Doses ranged from 0.625 to 5 mg/kg • Survival rates ranged from 28% at 0.625 mg/kg to 100% at 5 mg/kg (over a 7-day follow-up period) • No side effects of treatment were observed	Schuch et al. 2019
LysB4	*B. anthracis* Sterne 34F2 (1 × 10^7^ CFU/ml; i.n.)	Mouse	i.n.	• Lysin was administered i.n. at either 10 µg/head or 100 µg/head doses 6, 24, or 48 h post-infection • 14-day follow-up period • The higher lysin dose gave a 100% survival rate and no symptoms • The lower dose delayed the onset of deaths and gave approx. 50% survival rate	Park et al. 2018
PlyPH	*B. cereus* RSVF1 (2.5 × 10^6^ CFU/ml; i.p.)	Mouse	i.v.	• Lysin (3 mg/ml) was injected 10 min post-infection in 400 µl doses • 5-day follow-up period • Survival rate was 40% • No side effects were observed after administration of PlyPH alone	Yoong et al. 2006
PlyG	*B. cereus* RSVF1 (1 × 10^6^ CFU/ml; i.p.)	Mouse	i.p.	• 1 U corresponded to 1 µg of PlyG • 3-day follow-up period • After injection of 50 U of lysin in 0.5 ml dose 15 min post-infection, 68.4% of mice recovered fully • After injection of 150 U of lysin in 0.5 ml dose 15 min post-infection, 76.9% of mice recovered fully • No toxicity was detected after administration of PlyG alone	Schuch et al. 2002

*i.n.* intranasally, *i.v.* intravenously, *i.p.* intraperitoneally

The amount of bacteria inoculum was prepared to cause rapidly fatal illness and achieve the highest mortality (80–100%). In the control groups of mice treated with buffer instead of lysins, 86–100% mortality up to 7 days was observed. *B. cereus* RSVF1 applied intraperitoneally caused the most rapid deaths (100% mortality after 5 and 38 h for PlyG and PlyPH, respectively). The doses and the time of lysins administration used in the presented research were diverse. In PlyB, PlyG, and LysB4 studies, low-lysin-dose and high-lysin-dose groups of animals were compared. Survival rates increased proportionally with dosage enhancement. The maximum amount of micrograms of lysins resulted in 76.9% survival rate in the case of PlyG and 100% in the case of PlyB and LysB4. For PlyPH, a single dose was applied, and it reached a 40% survival rate. In the case of *Bacillus* administered intraperitoneally, the administration time of lysins ranged from 10 min to 1 h post-infection. LysB4 was delivered intranasally 6, 24, or 48 h after infection to reflect natural anthrax disease development and treatment. Efficient rescue and improvement of clinical signs after lysin therapy were observed. In control groups of mice, after administration of the lysins alone, no side effects were observed (no such data for LysB4) (Schuch et al. 2002, 2019; Yoong et al. 2006; Park et al. 2018). In PlyB research, its efficacy against *B. anthracis* was compared to that of the PlyG, revealing comparable results in both cases. Moreover, PlyG and PlyB in combination significantly enhanced efficacy against *B. anthracis* (Schuch et al. 2019).

## Multiple amino acid sequence alignment of the *B. anthracis *lysins’ CBDs

In contrast to the conservative EAD domains, CBDs exhibit significant diversity and thus determine the lysin specificity towards bacteria. Therefore, to facilitate a comprehensive comparison of these crucial regions, we performed a multiple sequence alignment of the CBDs’ amino acid sequences from the presented *B. anthracis* lysins. The alignment was generated using ClustalW with slow/accurate pairwise alignment, employing the BLOSUM weight matrix for proteins, and default parameters (https://www.genome.jp/tools-bin/clustalw). The AP50-31 lysin, possessing a monomodular structure without a CBD domain, was excluded from the comparison. Seventeen compared lysins, with their respective predicted CBD domain types, are listed in Table 3. The specific ranges of the CBD domains were sourced from the corresponding manuscripts where possible. When no ranges were specified, UniProt (https://www.uniprot.org/) and/or InterPro tools (https://www.ebi.ac.uk/interpro/) were employed to obtain the CBD domain types and ranges.

**Table 3:** The amino acid residue ranges of each *B. anthracis* lysin’s CBD, utilized for conducting the CBD multiple alignment, along with the identified CBD types

Lysin	aa residues	CBD domain type	Lysin	aa residues	CBD domain type	Lysin	aa residues	CBD domain type
PlyP56 PlyN74 PlyB PlyTB40 LysB4	186–252 206–275 206–275 201–244 186–252	SH3_b SH3_b SH3_b SH3_5 SH3_5	LysJ LysF Ply57 gp217 LysBC17 LysPW2	212–263 212–263 183–214, 254–306 171–237, 251–309 147–201, 220–281 175–233, 245–303	SH3 SH3 SH3b + SH3b SH3b + SH3b SH3b + SH3b SH3_3 + SH3b	PlyG PlyBt33 AmiBA2446 PlyPH PlyL Ply67	189–232 224–269 201-244 224-267 224–267 26–68	Amidase02_C Amidase02_C Amidase_C Amidase_C Amidase_C LysM

**Fig. 1:**
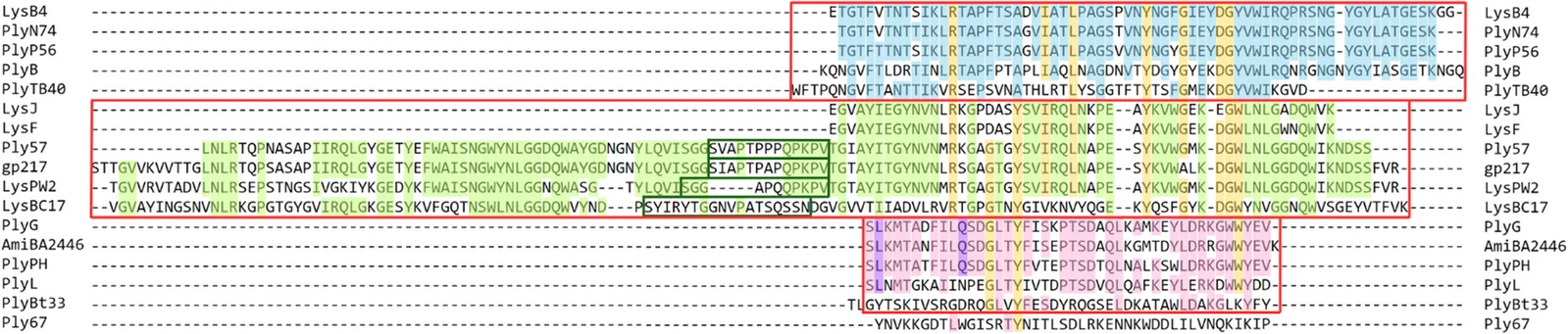
Multiple sequence alignment of the cell wall–binding domains of the 17 *B. anthracis* lysins. The alignment and the positioning of the particular domains were generated by ClustalW. Three distinct blocks, indicated by red frames, were proposed to encompass the most similar CBDs sharing analogous domain types, spanning from Block I at the top to Block III at the bottom of the alignment. Within each block, amino acid residues common to more than half of the aligned sequences are highlighted in blue, green, and pink. Yellow indicates the amino acid residues that are conserved across more than one block. Purple indicates the amino acid residues critical for the binding activity of PlyG to *B. anthracis* (Kikkawa 2007), which are repeated in the closely related lysins within the respective block. The sequences of the short linkers connecting two CBDs in lysins Ply57, gp217, LysPW2, and LysBC17 are marked by green frames

**Fig. 2:**
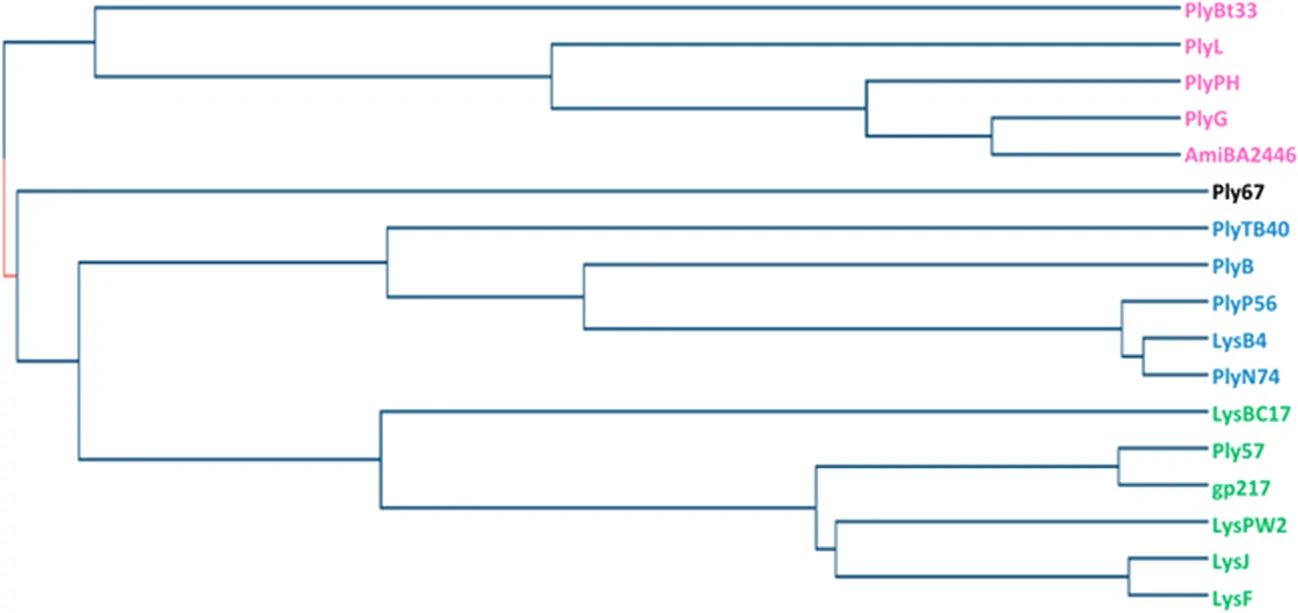
Phylogenetic tree of CBD domains. The amino acid sequence of each CBD domain was used to create a neighbor-joining tree without distance corrections (UniProt). Blue represents Cluster 1, green Cluster 2, and pink Cluster 3 of the CBDs domain

The multiple alignment of CBDs is depicted in Fig. 1. Among the 17 lysins’ CBDs, we were able to broadly group 16 of them into three main blocks. Block I included CBD domains of PlyP56, PlyN74, PlyB, PlyTB40, and LysB4. Bioinformatic analysis revealed highly conserved sequences in all these CBDs, each consisting of a single SH3_b or SH3_5 domain. Block II comprised the CBD domains of LysJ, LysF, Ply57, gp217, LysBC17, and LysPW2. Among these, four demonstrated a closely related double-CBD structure with a short linker between them, varying in length and amino acid composition. The ranges of these linkers’ sequences were determined using UniProt and subsequently verified through domain 3D modeling with SWISS-MODEL (https://swissmodel.expasy.org/interactive/gcmaSU/models/). The confirmed spans were marked with green frames in Fig. 1. 3D models of the predicted protein structures of LysJ and LysF lysins also suggest that they consist of two CBD domains (Nakonieczna et al. 2024) however, only one module (212–263 aa) has been verified (identified as the SH3 domain) and only these regions were included in the alignment. Block III encompassed PlyG, PlyBT33, AmiBA2446, PlyPH, and PlyL CBDs, all identified as Amidase02_C or Amidase_C domains. The remaining Ply67 could not be assigned to any of the blocks due to its dissimilarity. Its CBD was recognized as a LysM domain, a common small protein module involved in binding the peptidoglycan in bacteria. Additionally, employing the same sequences as input in the UniProt Align tool followed by the Trees building option, we generated a phylogenetic tree illustrating the relationships among the CBD domains of *B. anthracis* lysins (Fig. 2).

## Concluding remarks and prospects

The literature in recent years has provided limited descriptions of anthrax lysins active against *B. anthracis*. Although new anthrax phages are being reported, their lysins are often not produced or studied, as seen in the cases of phages Negev_SA, Tavor_SA, and Carmel_SA (Alkalay et al. 2018). In our work, we reviewed the enzymatic activity of all lysins from 41 known complete *B. anthracis* phage genomes (https://www.ncbi.nlm.nih.gov/). Among these, the majority (31 out of 41) exhibited amidase activity, suggesting a potential trend in *B. anthracis–*specific lysins. The remaining lysins included two with peptidase activity, one with lysozyme activity, and five whose enzymatic activities remain unverified.

We conducted a comprehensive literature search and compiled all lysins, to the best of our knowledge, that exhibit the capability to eliminate *B. anthracis* cells. Due to the different protocols employed for lysin activity assays in vitro and diverse ways of presenting the results, it is hard to compare the lysins featured in this review one to one and choose the best-acting one. Nevertheless, for certain lysins (AmiBA2446, PlyB, PlyBt33, LysBC17, LysB4, PlyP56, PlyN74, and PlyTB40), biochemical characterization, including thermal stability, pH range, or salt concentration impact, was also performed, which makes it easier to choose and adjust them to the desired purposes.

In addition to phage lysins, bactericidal enzymes include lytic enzymes encoded by bacteria, called autolysins. These two groups exhibit numerous advantageous properties, as they are quite similar in many respects (Mehta et al. 2013). Notably, a well-described autolysin with established activity against *B. anthracis*, not included in Table 1, is worth mentioning in this review. AmiBA2446 (NP844822) is an autolysin of bacterial origin, identified in the *B. anthracis* Ames strain A2012 (Mehta et al. 2013). This amidase (245 aa) had potent antibacterial activity against *B. anthracis* (ΔSterne, Sterne 34F2), *B. cereus* (ATCC 4342, Frankland and Frankland 1887 AL), and *B. thuringiensis* subsp. *kurstaki* ATCC 33,679 strains, which was measured spectrophotometrically. For all these strains except for *B. cereus* Frankland and Frankland 1887 AL, an almost 100% killing effect was observed (Mehta et al. 2013). Another interesting example of a bacteriolytic enzyme we can recall is Ply67 (ALN97746.1) whose activity was confirmed for *B. anthracis* endospores (Fu et al. 2020). Ply67 (217 aa) was identified in *Bacillus pumilus* phage BpSp. Unlike most phage lysins, this hydrolase does not have amidase/peptidase activity but is a spore cortex-lytic enzyme. Ply67 joins some features of phage lysins and cortex-lytic activity of GSLEs (germination-specific cortex-lytic enzymes) identified in *Bacillus* and *Clostridium* species. Its amino acid sequence exhibits 55% similarity to *Bacillus* phage AR9 endolysin and 40% homology to the *B. anthracis* GSLE, SleB (Fu et al. 2020). In the phage BpSp genome, there are two genes encoding hydrolases. A product of the gene *gp067*, Ply67, leads to the death of spores of *B. anthracis*, *B. cereus*, and *B. thuringiensis* rather than their germination, causing spore surface damage and their shrinking. The gene *gp019*, in turn, encodes *N*-acetylmuramoyl-l-alanine amidase, which could be the regular phage endolysin, but so far, it has not been described in detail (Yuan and Gao 2016).

Just a few *B. anthracis* lysins, namely PlyB, PlyG, PlyPH, and LysB4, have been examined in animal models, with all demonstrating satisfactory outcomes in mice (Table 2). Specifically, a lysin combination (the cocktail of PlyB and PlyG) was observed to enhance efficacy, potentially reducing therapeutic doses and treatment duration (Schuch et al. 2002). *Bacillus* strains used in the presented in vivo studies are well-established models of anthrax. Nevertheless, a more virulent strain is desirable due to the need to reflect infection and treatment accurately. Moreover, in most discussed studies, growing vegetative cells of *Bacillus* were used (spores were applied only in the LysB4 antimicrobial activity test). Application of *Bacillus* spores would be more suitable because of its expected usage in bioterrorism attacks (Park et al. 2018). Even though the mouse model is approved as a standard, it cannot fully simulate an infection of the human body due to the differences in anatomy, physiology, and the mechanism of action of the immune system. Mice exhibit features such as a weak or absent cough reflex, a lack of bronchioles, and a proportionally larger nasal surface area with less airway branching compared to humans. Moreover, substantial differences exist in the functions and effectors of murine and human immune systems (Welkos et al. 2016). Therefore, another animal model is recommended to test the efficacy of lysins. Primates seem more appropriate as they are most related to the human species (Park et al. 2018). Also, the discussed studies did not use an aerosol challenge model, which is desirable since the inhalational route of administration reflects the natural transmission of anthrax bacilli and is most likely to be applied in potential bioterrorism incidents (Loving et al. 2007).

The comparison of amino acid sequences within the variable cell-binding domains of the 17 lysins revealed notable similarities and differences, allowing us to categorize 16 of them into three main groups (Fig. 1). The sequence of the remaining Ply67 lysin stands out due to its low similarity level. This discrepancy appears consistent with Ply67 characterization as a partially cortex-lytic protein, distinguishing it distinctly from the other lysins. In the proposed Block I and II, the SH3 domain was identified as a common CBD type. SH3b domain(s) are widely utilized by many lysins as their CBD and are found in a variety of proteins with enzymatic activity. These domains typically adopt the characteristic SH3 β-barrel fold, consisting of 5–7 β-strands. The folds often form two tightly packed antiparallel β-sheets joined by the linker (Broendum et al. 2018). The closer relatedness of the lysins from these two blocks is also evident in the phylogenetic tree, where these two blocks emerge from a single branch (Fig. 2). Amidase02_C module, in turn, appears only in phages that infect A1α or A1γ bacterial peptidoglycan chemotype; therefore, the occurrence of this domain is not surprising since the peptidoglycan A1γ-chemotype is widely distributed among Bacillaceae (Archibald et al. 1993; Vázquez et al. 2021).

Two amino acid residues in the PlyG CBD that were reported as critical for the binding to *B. anthracis*, L190 and Q199 (Kikkawa et al. 2007), are repeated in the most closely related lysins found within Block III (highlighted in purple in Fig. 1). This homology observed among the binding domains of PlyG, PlyPH, and AmiBA2446 may contribute to a similarity in their host range and thereby the specificity of these enzymes. Both, PlyG and PlyPH, demonstrate specific lytic activity exclusively against *B. anthracis* strains and a surrogate strain, *B. cereus* ATCC 4342 (Table 1). Additionally, AmiBA2446 exhibits antimicrobial activity against *B. thuringiensis* subsp. *kurstaki* ATCC 33,979 which shares a close genetic resemblance with *B. anthracis* (Mehta et al. 2013). In contrast, PlyL has only one of these critical amino acid residues, L190, which potentially accounts for variations in its host range, displaying notable activity against *B. cereus, B. subtilis*, and *B. megaterium* but lower against *B. anthracis* Sterne 34F2 (Low et al. 2005). However, given the differences in available data regarding the bacterial host ranges of all described lysins, direct comparisons are challenging, making it difficult to unequivocally determine whether the proposed blocks of lysins correlate with their specific host ranges.

For many phage researchers, there is a perspective that phages and their lytic enzymes can, in the future, completely replace antibiotics, especially against multi-drug-resistant bacteria (Mehmood Khan et al. 2023). Due to certain obstacles to lysin administration to patients, like possible proteolysis, methods of safe and effective delivery systems, including encapsulation, are being developed (Gondil and Chhibber 2021). Furthermore, advancements in the molecular engineering of lysins and their domains offer promising prospects for enhancing their efficacy (Seijsing et al. 2018). This suggests that native or engineered lysins could potentially serve as an alternative treatment for anthrax in animals or humans, especially in situations where the use of antibiotics is restricted or undesirable.
